# Genetic diversity and population structure of wild and cultivated *Crotalaria* species based on genotyping-by-sequencing

**DOI:** 10.1371/journal.pone.0272955

**Published:** 2022-09-01

**Authors:** Joshua Kiilu Muli, Johnstone O. Neondo, Peter K. Kamau, George N. Michuki, Eddy Odari, Nancy L. M. Budambula

**Affiliations:** 1 Department of Biological Sciences, University of Embu, Embu, Kenya; 2 Institute for Biotechnology Research, Jomo Kenyatta University of Agriculture and Technology, Nairobi, Kenya; 3 Department of Life Sciences, South Eastern Kenya University, Kitui, Kenya; 4 The African Genomics Centre and Consultancy, Nairobi, Kenya; 5 Department of Medical Microbiology, Jomo Kenyatta University of Agriculture and Technology, Nairobi, Kenya; National Cheng Kung University, TAIWAN

## Abstract

*Crotalaria* is a plant genus that is found all over the world, with over 700 species of herbs and shrubs. The species are potential alternative food and industrial crops due to their adaptability to different environments. Currently, information on the genetic diversity and population structure of these species is scanty. Genotyping-by-sequencing (GBS) is a cost-effective high-throughput technique in diversity evaluation of plant species that have not been fully sequenced. In the current study, *de novo* GBS was used to characterize 80 *Crotalaria* accessions from five geographical regions in Kenya. A total of 9820 single nucleotide polymorphism (SNP) markers were obtained after thinning and filtering, which were then used for the analysis of genetic diversity and population structure in *Crotalaria*. The proportion of SNPs with a minor allele frequency (maf) > = 0.05 was 45.08%, while the Guanine-Cytosine (GC) content was 0.45, from an average sequence depth of 455,909 reads per base. The transition vs transversion ratio was 1.81 and Heterozygosity (He) ranged between 0.01–0.07 in all the sites and 0.04 to 0.52 in the segregating sites. The mean Tajima’s D value for the population was -0.094, suggesting an excess of rare alleles. The fixation index (Fst) between the different populations based on the Wright Fst (1943) ranged from 0.0119 to 0.066 for the Eastern-Western and Nairobi-Western populations. Model based techniques of population structure analysis including structure, k-means and cross-entropy depicted eight clusters in the study accessions. Non-model based techniques especially DAPC depicted poor population stratification. Correspondence Analysis (CA), Principal coordinate analyses (PCoA) and phylogenetic analysis identified a moderate level of population stratification. Results from this study will help conservationists and breeders understand the genetic diversity of *Crotalaria*. The study also provides valuable information for genetic improvement of domesticated species.

## Introduction

*Crotalaria L*. is a plant genus comprising 702 herb and shrub species which are widely distributed especially in the Southern Hemisphere. Subtropical Africa and Madagascar are the primary centres of diversity for these species, with approximately 543 species [1]. *Crotalaria* species occupy diverse habitats but are mostly found in open grasslands, forests edges and road sides [2]. These species are used for a variety of purposes all over the world, including their use as vegetables, use in control of nematodes, medicinal applications, their use as green manure and in the control of *Striga hermonthica* (Delile) Benth. [3]. With the world population growing and food sources diminishing, there is need to diversify food sources [4]. Some of the potential means to this end include exploitation of under-utilized and semi-domesticated *Crotalaria* species whose potential not only as food but also industrial crops are immense. Only about 15 *Crotalaria* species are eaten around the world making it important to prospect within the genus for more edible varieties, as the plants are highly adaptable and relatively cheap to produce [3]. To achieve this, there is need to identify the close relatives of domesticated *Crotalaria* species and determine their inter-species diversity as a prerequisite step. Diversity estimates and relationship studies on edible *Crotalaria* species will make it possible to develop breeding programs to improve the already domesticated species through interspecific hybridization. Currently, genetic diversity studies of the genus *Crotalaria* based on molecular data are scanty. Only two studies have been reported, one involved the use of the internal transcribed spacer (ITS) sequence data and the other used start codon targeted (SCoT) markers [5, 6].

In nature, *Crotalaria* species have different levels of ploidy, thereby increasing the complexity of the entire genus. Although the chromosome number for most species is 2n = 16, in some species such as *Crotalaria incana* L., the ploidy number is 2n = 14, while polyploidy has been reported in other species such as *Crotalaria paulina* Schrank and *Crotalaria stipularia* Desv [7, 8]. The presence of bisexual flowers makes some species in the genus self-compatible with a low degree of crossing while others are cross pollinated with a moderate degree of self-pollination [9]. Interspecific hybridization among these species could play an important role in *Crotalaria* improvement by introducing novel traits from wild to domesticated species. However, before this can be achieved, there is need to determine species relatedness by establishing the genetic distances between them.

Plant diversity assessment is a crucial stage in breeding, conservation planning and evolution research [10]. To assess diversity there is need to identify markers albeit morphological, biochemical or molecular, especially in plant species that have not been widely domesticated. In the *Crotalaria* genus, morphological parameters are relatively uniform and therefore might not be the optimum tool for morphological analysis [11]. This necessitates the need for DNA based markers such as single nucleotide polymorphisms (SNPs). Apart from being abundant in plant species, SNPs also have a wide range of sequence variations within plant genomes [12]. This makes them markers of choice in breeding and research on different aspects of plants like quantitative trait loci (QTL) mapping, population structure analysis, genome wide association studies (GWAS), evolutionary studies among others [13]. SNP identification has become faster and cheaper thanks to the advancement of high-throughput sequencing techniques, particularly next generation sequencing (NGS), with GBS being one of the current methods in use. Furthermore, GBS technology can be used to sequence plant species which have no reference genome [14]. Plant breeding mostly aims at impacting adaptability to biotic and abiotic stress in domesticated crops. To achieve this, marker assisted selection (MAS) techniques are used by breeders. To identify genes associated with any quantitative trait, there is need for prior studies to be done on the phenomic trait as well as to have sequencing data for association mapping [15]. After the identifying the QTLs associated with certain traits, advanced pre-breeding techniques can be used to transfer the genes to crops of interest [16]. Genomic prediction is a modern tool that is used to identify genes associated with specific traits. The process involves phenotype prediction from genetic markers through modelling [17]. As yet, few studies have been done to ascertain the morphological and molecular variability in *Crotalaria* species. The studies done so far include those of [1, 2, 5, 6, 18–24]. The use of markers in some of these studies was minimal. Where involved, the markers were either tested for their adaptability in *Crotalaria* species or to determine the relationship of involved accessions. Hence, most of these molecular markers cannot be extensively used for breeding purposes such as the identification of important QTLs for the improvement of *Crotalaria*. The current study involved the use of NGS for identification of SNP markers in *Crotalaria* and is the first of its kind in the genus.

## Materials and methods

### Study site and sampling

Samples for the study were collected using a subjective non-probability sampling technique [25]. The process began with a reconnaissance survey to establish regions where *Crotalaria* species are grown. Farmers were then selected in these regions and interviewed to determine the socio-economic impact of *Crotalaria* farming among their communities, and samples collected for diversity studies. Samples were collected from five regions in Kenya with varying rainfall and temperatures, namely; Nairobi, Western, Nyanza, Eastern and Rift Valley. The five sampled regions fall into three climatic regions of agricultural importance in Kenya which are the temperate zone, the coastal strip and the hot and dry zones [18]. This brought together *Crotalaria* germplasm cordially provided by the Genetic Resources Research Institute of Kenya (samples with the prefix GBK) and farmer-held accessions totaling to 80 samples (S1 Table). These samples represent 21 different *Crotalaria* species from the five regions, a relatively adequate population for a GBS based diversity study. A research permit for purposes of field site access and sample collection was issued by the National Commission for Science Technology and Innovation in Kenya (NACOSTI-Kenya) and verbal consent was sought from the farmers at the time of seed collection. No minors were involved in the study.

### DNA isolation

One gram of each *Crotalaria* leaf sample was transferred into 2ml Eppendorf tubes containing 2 steel beads then immediately immersed in liquid nitrogen. The tubes were then vortexed using the vortex-genie^®^ 2T mixer at maximum speed and the leaf samples ground into fine powder. To the fine powder, 500μl of 3% cetyl trimethylammonium bromide (CTAB) extract buffer pH 5 with 0.2% β-mercaptoethanol and 1% polyvinylpyrrolidone were added and incubated at 65°C for 30 minutes in a water bath. After incubation, the tubes were filled with 500μl chloroform:isoamyl alcohol (24:1) and gently mixed until a homogenate mixture was obtained. The mixture was then centrifuged at 13000 revolutions per minute (RPM) at 4°C for 10 minutes. Using a pipette, the upper aqueous layer was carefully aspirated into clean tubes without the inter-layer or the organic layer. A volume of isopropanol that was two thirds of the aspirated liquid was added to the new tubes and mixed, then incubated at -20°C for 1 hour. The mixture was centrifuged at 13000 RPM at 4°C for 10 minutes following incubation, and the supernatant was carefully discarded to avoid losing the formed pellet. After centrifuging, 70% ethanol (500μl) was added to the tubes and tapped gently to dislodge the DNA pellet, and the supernatant discarded. This washing step was repeated twice and the tubes placed on a clean paper towel for an hour to dry the pellet. The dry DNA pellet was dissolved in 100μl nuclease free water and stored at -20°C prior to shipping to Hong Kong [26].

### Genotyping by sequencing

GBS analysis was carried out at the BGI Tech Solutions Ltd in Hong Kong. Individual digestion of all DNA samples was done using *ApeKI* restriction endonuclease, which recognizes a five base pair palindrome sequence (G/CWGC). The digested DNA was ligated to barcoded primers to create a GBS sequencing library. The adaptor information and library kit used to create the GBS library were as described by [27]. This was followed by sequencing using Illumina HiSeq2500.

### Data preparation

Obtained FASTQ sequences were subjected to the FASTX-tool kit (v 0.0.13) for quality trimming and filtering, with the set parameters–q minBaseQ>20. After filtering and trimming, quality assessment of the obtained sequences was done using FastQC (v 0.11.4) to select sequences with a Phred score above Q30. Pass quality sequencing reads were then assembled *De novo* using the NGSEPcore software version 4.1.0 [28]. PLINK was used to convert the SNP data into PED and MAP format [29]. The vcftools flags—depth and—site-depth were used to set read depth per individual and per SNP [30]. Binary files (BED, RAW and BIM) were generated using PLINK from PED and MAP files [29], that is, using the flags—make-bed,—recode A,—chr-set 95, and allow-extra-chr.

### Data management

SNP data management and analyses were performed in R-4.0.4 [31] using wrapper functions of the R package SambaR (github page: https://github.com/ mennodejong1986/ SambaR). Using the ’read.PLINK’ function from the R package adegenet-2.1.3, the data was imported into R and stored in a genlight object [32, 33]. The data was filtered using the function ’filterdata’ of the R package SambaR, with the parameters set as; with indmiss = 0.95, snpmiss = 0.15, min_mac = 2, dohefilter = TRUE and min_spacing = 500. This discarded all the samples with missing data of more than 95% (indmiss = 0.95), SNPs with more than 15% missing data which was averaged over samples which passed the indmiss threshold (snpmiss), SNPs containing only one copy of the minor allele (min_mac), SNPs with heterozygosity levels which were potentially indicative of paralogs (dohefilter) and to ensure that the minimum distance between adjacent SNPs was 500bp when thinning the data (min_spacing). After filtering 80 out of 80 individuals (3–34 per population) and 9820 out of 434274 SNPs were retained. This filtered dataset were used for selection analyses. Thinning retained the dataset at 9820 SNPs. This filtered and thinned dataset were used for structure analyses. The GC-content of the retained dataset equaled 0.45 and the ’transversion vs transition’-ratio equaled 0.64.

### Structure analyses

Correspondence analyses (CA) were performed using the function ’dudi.coa’ of ade4-1.7.16 R package [34, 35]. Data was imputed per SNP/individual by calculating genotype probabilities from population specific minor allele frequencies’. Principal coordinate analyses (PCoA) were done using the ’pcoa’ function of ape-5.4.1 R package [36] on distance matrices containing three different measures of genetic distance: Nei’s genetic distance, calculated with the function ’stamppNeisD’ of StAMPP-1.6.1 [37]; Hamming’s genetic distance, computed with the ’bitwise.dist’ function of poppr-2.9.0 [38] and Π (pairwise sequence dissimilarity), calculated with the function ’calcpi’ of SambaR [39]. Principal component analyses (PCA) were done using the ’snpgdsPCA’ function of the SNPRelate-1.24.0 R package [40]. Discriminant analysis of principal components (DAPC) analyses were performed using the function ’dapc’ of the adegenet-2.1.3 R package [32, 33], both with and without prior population assignment. Using a stratified cross validation technique with variable principle components (PCs), PCs with the least mean square error and the most success were evaluated and retained to determine genetic clustering patterns in *Crotalaria*.

Admixture coefficients were calculated using the functions ’obj.snmf’ and ’Q’ of the LEA-3.2.0 R package [41]. Alpha was set to 10, number of iterations to 200 and tolerance to 0.00001. Ancestry coefficients were calculated with the software Admixture-1.3 [42] and plotted using the ’plotstructure’-function of SambaR. Using admixture, samples were assigned to a cluster (K) based on the ancestry fraction that was estimated for every individual. The best choice of the number of clusters was once again tested using five separate runs. To detect outlier SNP loci from the 9820 SNPs genotyped for the 80 *Crotalaria* individuals, PCAdapt and OutFLANK R packages were used. PCAdapt is based on PCA analysis while OutFLANK is based on atypical values of Fst [43, 44]. The Bayesian clustering methods (STRUCTURE, LEA, and TESS) were used to determine the patterns of population structure. Individuals were assigned to sampling localities and genetic admixture levels tested by genetic clusters without using priori sampling information. TESS was also used to explore spatial population structure from both genotypic and geographical information as previously explored by [45]. To determine the optimal number of ancestral populations, a cross-entropy method was explored using the *snmf* function in LEA. Phylogenetic analysis was done using the MEGA software version 11.0.10 by the neighbor-joining method [46] and BEAST version 2.4.5 [47]. In MEGA, the Maximum Likelihood method and Tamura-Nei model were used to infer the evolutionary history. Application of the Neighbor-Join and BioNJ algorithms to a matrix of pairwise distances which had been estimated using the Tamura-Nei model, and by selecting the topology with superior log likelihood value, initial trees for the heuristic search were generated. In BEAST, the five populations were all used as a priori designated clusters, and the analysis run for 1,000,000 generations, with sampling being done every 1,000 generations as guided by [48].

### Genetic distance analyses

The function ’stamppNeisD’ of the StAMPP-1.6.1 R package was used to calculate Nei’s genetic distance [37]. Genome wide ’Weir & Cockerham 1984’ Fst estimates (for all pairwise population comparisons) were calculated using the function ’stamppFst’ of the StAMPP-1.6.1 R package as well as their associated Pearson’s r and p-values [37]. Locus specific Fst estimates according to Wright (1943), Nei (1977), and Cockerham & Weir (1987) for all pairwise population comparisons were calculated with the functions ’runWrightFst’, ’locusNeiFst’, and ’locusWCFst’ of the R package SambaR. Relatedness between samples was calculated using the softwares GCTA and plotted using SambaR functions.

### Genetic diversity analyses

Linkage disequilibrium (LD) estimates were calculated using PLINK (-genome—r2—ld-window-kb 1000000—ld-window -r2 0). Hardy-Weinberg Equilibrium (HWE), (2D) folded site frequency spectra (SFS), Tajima’s D and genome wide heterozygosity analyses were executed using the function ’calcdiversity’ of the R Package SambaR. Population specific SFS vectors were generated using the function ’getfoldedsfs’ of the R package SambaR, which bins SNPs into classes based on how many copies of the minor allele they possess and then calculates the size of each bin (number of SNPs within each bin). Genome wide He (genomeHe) was calculated for each sample using the formula: genome He = (He_seg * N_seg)/N_total), where: N_seg: is the number of segregating sites within the population to which the individual under investigation belonged, He_seg: is the fraction of heterozygous sites for those segregating sites within the examined individual and N_total: is the total length of all polymorphic and monomorphic sequenced sites that passed the filter parameters.

## Results

### General characteristics of GBS in *Crotalaria*

The germplasm collection of 80 *Crotalaria* samples from different regions in Kenya was successfully sequenced using the GBS technology to yield information on the number of reads in millions, number of bases and the percent guanine-cytosine content (S2 Table). After filtering the raw reads, 428.16 M clean reads were obtained, ranging from 0.22 M to 14 M reads, which averaged to 5.35 M reads per sample. From the sequencing data, high base calling accuracy (Q scores) was obtained, with most of the reads scoring Q30 and above. The quality value 20 (Q20) ranged from 98.86 to 99.14 while quality value 30 (Q30) ranged from 97.05 to 97.75, suggesting a relatively high base call accuracy for each sample. The GC content ranged from 43.97–53.14%. The assembly of reads resulted in 2,657,824 clusters from a total of 428,191,702 reads in the 80 samples. From the 2,657,824 clusters, 862 were large (>8000) while 2,361,293 were small (<80), with totals of 52,678,737 reads in the large clusters and 38,717,104 reads in the small clusters. The proportion of total reads to the clustered reads in all the 80 samples used in the study are shown in S3 Table. The mean clustering ratio was 0.7, with a minimum of 0.44 and a maximum of 0.88.

### Single Nucleotide Polymorphism (SNP) diversity

A total of 434,274 polymorphic SNP sites from the 80 accessions were identified. The mean sequence depth averaged at 455,909 reads per base. From the 434,274 SNPs, the percentage of SNPs with maf > = 0.05was 73.85%, the GC content was 0.51 and the transition vs transversion ratio 1.58. The multi-locus heterozygosity (MLH) was 0.036while the standardized multilocus heterozygosity (sMLH) was 0.970 for the entire population. The individual MLH and sMLH are as depicted in S2C and S2D Fig. The SNPs were filtered under the conditions of maf > = 0.05, missing individuals/indmiss (0.95), missing SNPs/snpmiss (0.15), minimum minor allele count (2) and a min spacing of 500. After filtering, all the 80 accessions were retained. However, the total number of SNPs retained reduced to 9,820. The percentage of SNPs with maf > = 0.05 after filtering reduced to 45.08%, the GC content reduced to 0.45 while the transition vs transversion ratio increased to 1.81. The total genotyping rate in the 80 individuals was 0.33893. The nature of transition vs transversion ratios for both minor and major alleles before and after filtering are as depicted in S1 Fig. The most transversed/transited alleles were G/A, C/T, A/G, and C/T. Heterozygosity (He) ranged from 0.01 to 0.07 in all the sites and 0.04 to 0.52 in the segregating sites (S2A and S2B Fig). Among the 9820 SNP loci, the mean minor allele frequency (maf) and the average pairwise difference among individuals (Π) averaged 0.07 and 0.13 respectively. Of the 9,820 segregating sites in the 80 accessions, there were 3942 private alleles distributed across all the five populations, with the Nairobi population having a majority (3,628) of the private alleles, followed by the Western population (182), Rift Valley (107), Nyanza (24) and the Eastern population having only one. The maf in all the sites for the different populations was 0.027, 0.109, 0.051, 0.048 and 0.035 for Eastern, Nairobi, Nyanza, Rift Valley and Western populations respectively, and 0.246, 0.119, 0.110, 0.177 and 0.096 at the segregating sites for the same populations respectively.

### Diversity and divergence of Kenyan *Crotalaria* germplasm

**Fig 1:**
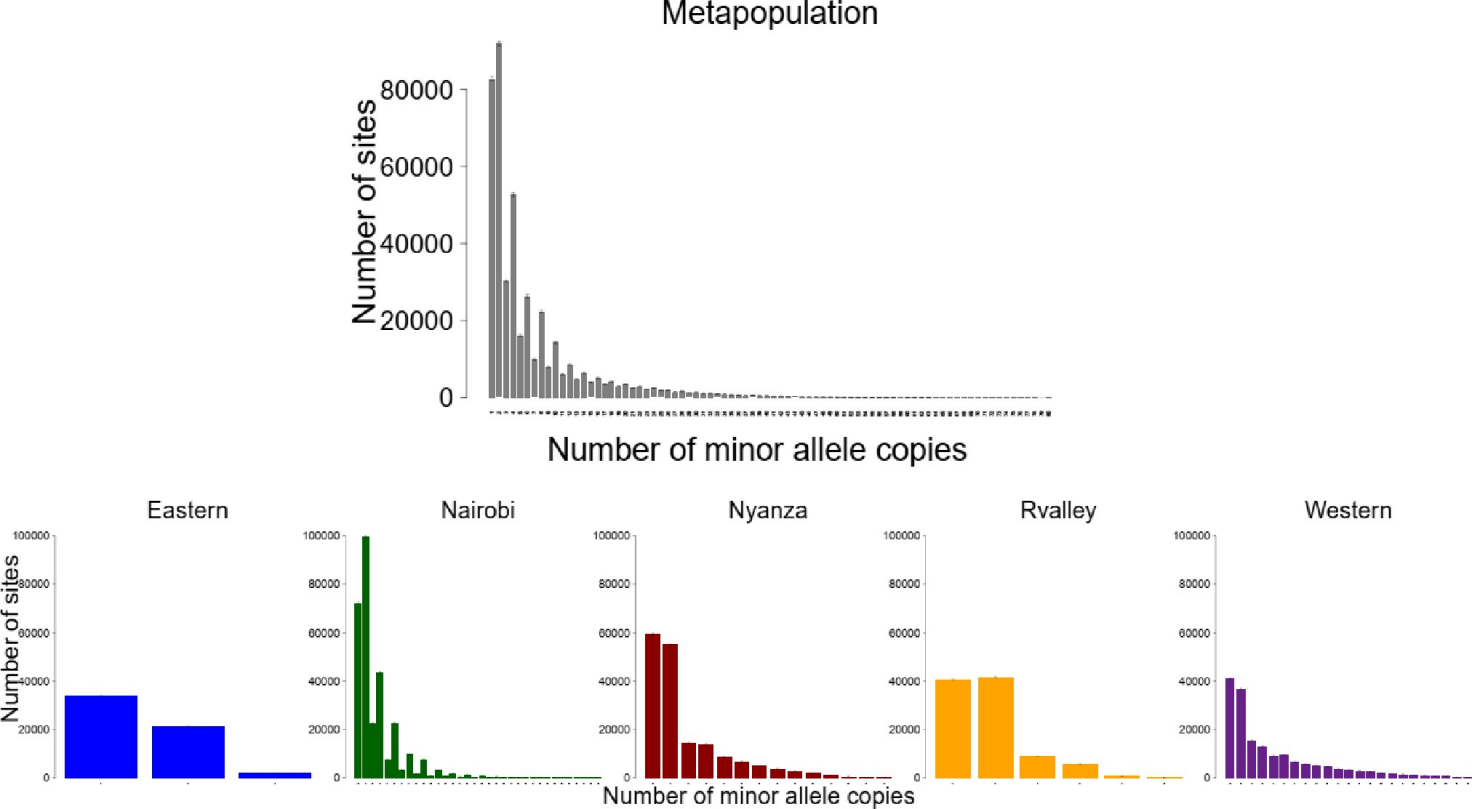
LD patterns observed in *Crotalaria* SNP data. a) LD box plot showing ranges of r^2^ values in the different populations, b) LD decay (in kb) in the SNP data set of 80 *Crotalaria* samples.

**Fig 2:**
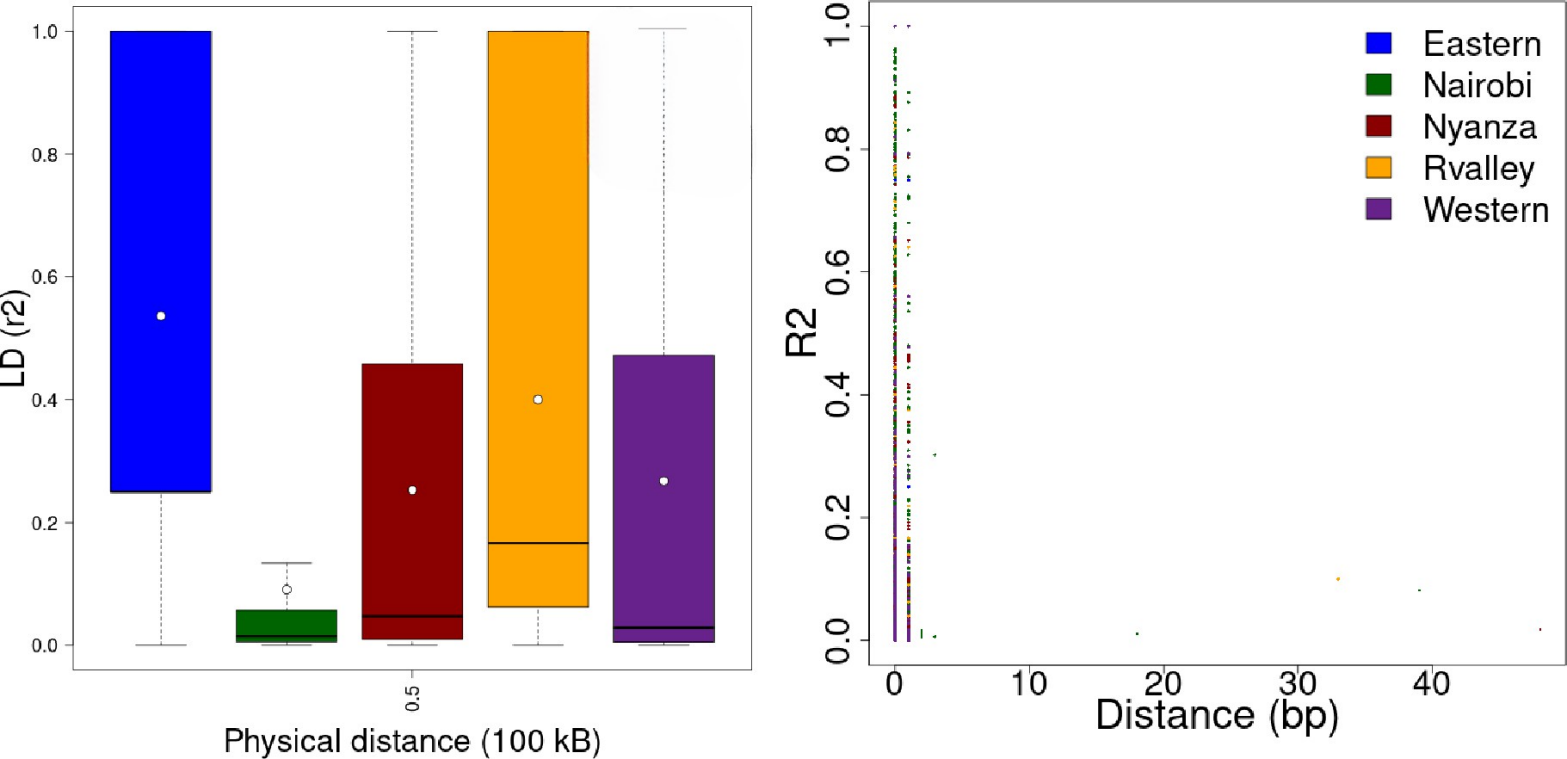
SFS bar plots based on the SNP data of 80 Kenyan *Crotalaria* samples.

The Π values for the different *Crotalaria* populations in this study were 0.05 for the Eastern population, 0.18 for Nairobi, 0.08 for both Nyanza and the Rift Valley populations and 0.06 for the Western population. These observed Π values differed from the expected values of 437.2, 2,162.9, 873.9, 788 and 631.8 for the Eastern, Nairobi, Nyanza, Rift Valley and Western populations respectively (S4 Table). The scaled Watterson estimator for the different populations was 0.057 (Eastern), 0.285 (Nairobi), 0.129 (Nyanza), 0.108 (Rift Valley) and 0.086 (Western). Based on the estimates of population genetic parameters Π and Watterson θ, all the Tajima’s D values for the different populations were negative and inversely proportional to the number of mutations (S), with a mean Tajima’s D value for the population being -0.094. According to the generated site frequency spectra (SFS) bar plots, the Nairobi population had the highest distribution of minor alleles across the polymorphic sites (100,000) while the Eastern population had the least (38,000) distribution of minor alleles in the polymorphic sites (Fig 1). Based on the computed HWE test score, the entire *Crotalaria* population under study was not in Hardy-Weinberg equilibrium, since the tabulated locus specific HWE chi squared score (20) was higher than the critical value (3.84). However, the Rift valley and Eastern sub-populations were in Hardy-Weinberg equilibrium (S3 Fig). Linkage disequilibrium calculations for all possible combinations (r^2^) of the 9820 SNPs revealed expected mean r^2^ values of 0.55, 0.4, 0.28, 0.27 and 0.1 for the Eastern, Rift Valley, Western, Nyanza and Nairobi *Crotalaria* populations (Fig 2A). These values imply a moderate to low predictability of SNP alleles in the involved *Crotalaria* populations. Further, the plotted LD scatter revealed a fast decay (Fig 2B).

**Fig 3:**
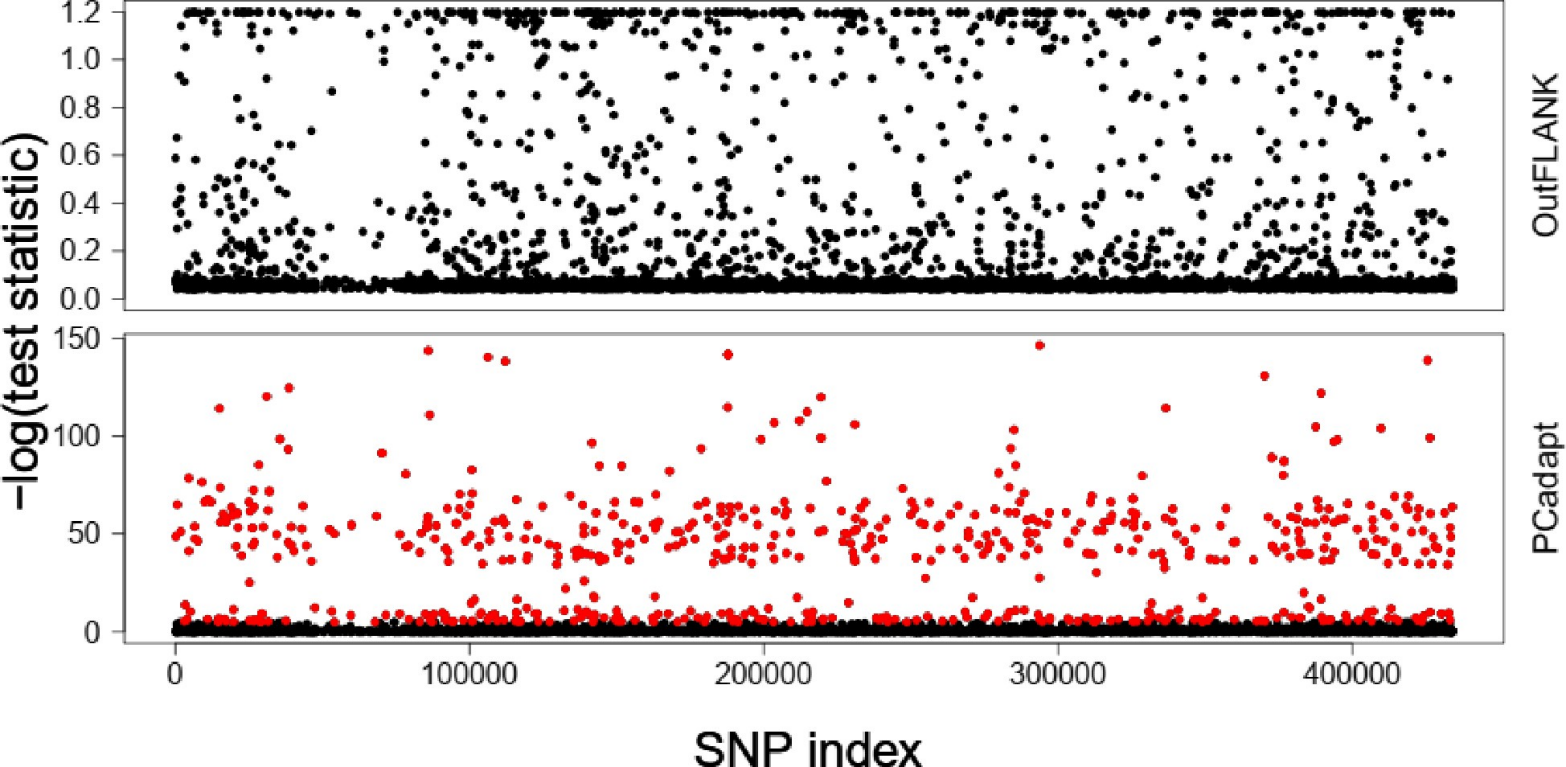
Putatively adaptive and neutral SNPs separation based on the F_ST_ OutFLANK and PCadapt techniques. From the 9820 SNP loci, PCadapt identified 649 SNPs (red circles) while F_ST_ OutFLANK did not identify any outlier SNPs. The remaining SNPs (black dots) were considered as putatively neutral loci.

As a measure of genetic divergence and inferred genetic clusters, the fixation index (Fst) between the different populations based on different Fst tabulation methods and their associated P values were considered (Table 1). The pairwise comparison of the different Fst values revealed relatively moderate to minimal population differentiation that can be accounted for by population structure. However, high Fst values were observed in population comparisons involving both domesticated and cultivated accessions such as the Nairobi-Western and Nairobi-Nyanza populations, while low Fst values were observed in population comparisons involving either non-domesticated accessions such as Eastern-Rift Valley or populations with highly domesticated accessions such as Nyanza-Western. Based on the calculated p values, there was no significant differentiation between Nyanza-Rift Valley, Nyanza-Western, Eastern-Rift Valley and Eastern-Nyanza *Crotalaria* populations, while all other comparisons revealed highly significant population differentiations. Based on Wrights (1943) Fst tabulation, there was moderate genetic drift between the Eastern-Nairobi (0.0233), Rift Valley-Western (0.0221), Nyanza-Rift Valley (0.0244) and Nairobi-Rift Valley (0.0334) *Crotalaria* populations. However, there was greater genetic drift between the Nairobi-Nyanza (0.0427) and Nairobi-Western (0.0666) populations while there was minimal genetic drift between all other populations. Clustering using the 9,820 putative SNP loci revealed little genetic differentiation among the different *Crotalaria* samples. Therefore, OutFLANK and PCAdapt approaches were applied to identify the putative adaptive *Crotalaria* loci under local selection pressure. PCAdapt and OutFLANK detected 649 and 0 outlier SNPs as putatively adaptive loci under divergent selection (Fig 3). The remaining SNPs were considered as putatively neutral loci. This suggests that most of the outlier SPS detected were under balancing selection, indicating that geographical isolation among the sampled sub-populations alone might not be enough to explain the observed population stratification.

**Table 1:** Population differentiation represented by different fixation index (Fst) estimates on Kenyan *Crotalaria* species.

Populations	Nei D	Reynalds Weir Cockerham	Rodgers	Provesti	Nei D stampp	Weir Cockerham	Wright	Weir Cockerham pvalue	Pearson r
Eastern-Nairobi	0.3098	0.8834	0.3307	0.3307	0.049	0.076	0.0233	0	0.31
Eastern-Nyanza	0.2414	0.8894	0.2673	0.2673	0.01	-0.0397	0.0163	1	0.25
Eastern-Rift Valley	0.18	0.8721	0.1887	0.1887	0.009	-0.1124	0.0169	1	0.46
Eastern-Western	0.2166	0.876	0.2321	0.2321	0.009	0.0315	0.0119	0	0.32
Nairobi-Nyanza	0.4239	0.899	0.3944	0.3944	0.035	0.1372	0.0427	0	0.11
Nairobi-Rift Valley	0.3544	0.8916	0.3514	0.3514	0.04	0.0996	0.0334	0	0.22
Nairobi-Western	0.4527	0.9009	0.397	0.397	0.046	0.2298	0.0666	0	0.02
Nyanza-Rift valley	0.2517	0.8898	0.2747	0.2747	0.009	-0.0031	0.0244	0.97	0.24
Nyanza-Western	0.2069	0.86	0.217	0.217	0.003	-0.0006	0.0125	0.85	0.36
Rift Valley-Western	0.2153	0.8721	0.2343	0.2343	0.009	0.0528	0.0221	0	0.35

### Genetic structure and phylogenetic analysis

**Fig 4:**
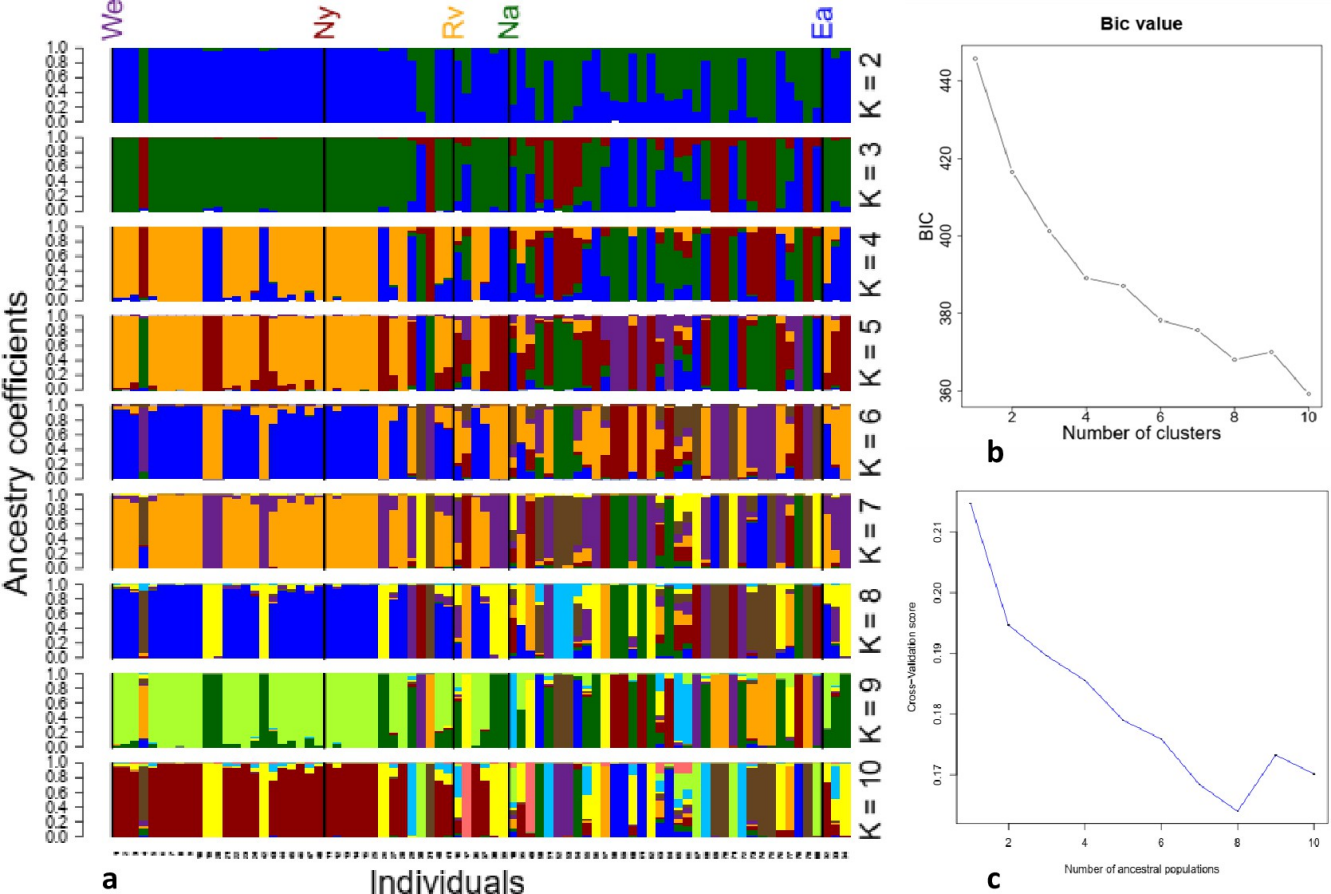
Population structure analyses of 80 *Crotalaria* accessions based on the GBS-SNP genotyping. **(a)** The stacked bar plot generated for the K values = 2–10. **(b)** The number of cluster (K) values as plotted using the Bayesian information criterion (BIC). (**c**) Cross-entropy plot with clusters (K) = 1 to 10.

Eight genetic clusters (K = 8) efficiently summarized the patterns of variation in the data, according to population structure analysis utilizing parametric and nonparametric (structure and k-means) approaches. The Bayesian Information Criterion (BIC) model established that 10 clusters (K) were required to control for population structure. The computed a score value (0.19) revealed a weak discrimination, while the stratified cross-validation revealed that the lowest mean square error was observed with 10 PCs. However, this K value had a lot of outliers hence K = 8 would be more appropriate. The number of clusters (K) was plotted against the BIC value to determine the most suited value of K. The plot revealed that the BIC value continually decreased from K = 1 up to K = 8, before increasing slightly. However, the lowest BIC value was observed at K = 10 (Fig 4B). A cross-entropy validation plotted against the number of ancestral populations in TESS revealed an optimum of eight ancestral populations (Fig 4C). However, cluster assignment maps obtained from TESS did not reveal a clear differentiation among the five sampled populations. Using admixture 1.3, at K = 2, all the accessions were distributed into two groups. Most of the accessions from the Nairobi region had mixed ancestry while those from Eastern, Western and Nyanza had relatively pure ancestry (Fig 4A). At K = 3, three groups were observed, with all the populations having mixed ancestry. All the groups observed at K = 3 were also observed at K = 4–10. Bayesian population assignment using 50 to 566 most informative SNPs revealed a decrease in population stratification as more SNPs were considered for population assignment. Considering 566 SNPs, most individuals were assigned entirely to a single population, with most samples being assigned to the Nairobi and Western populations while only two were assigned to the Nyanza population (S5A Fig). Using TESS, a geographical structure was identified, from which four distinct clusters were observed. TESS assigned most of the samples to the Nyanza population by ancestry at k = 5, and one sample each to Eastern and Rift Valley populations (S4C Fig). LEA q matrix also identified some level of population stratification with overlaps and mixed ancestry (S4B Fig).

**Fig 5:**
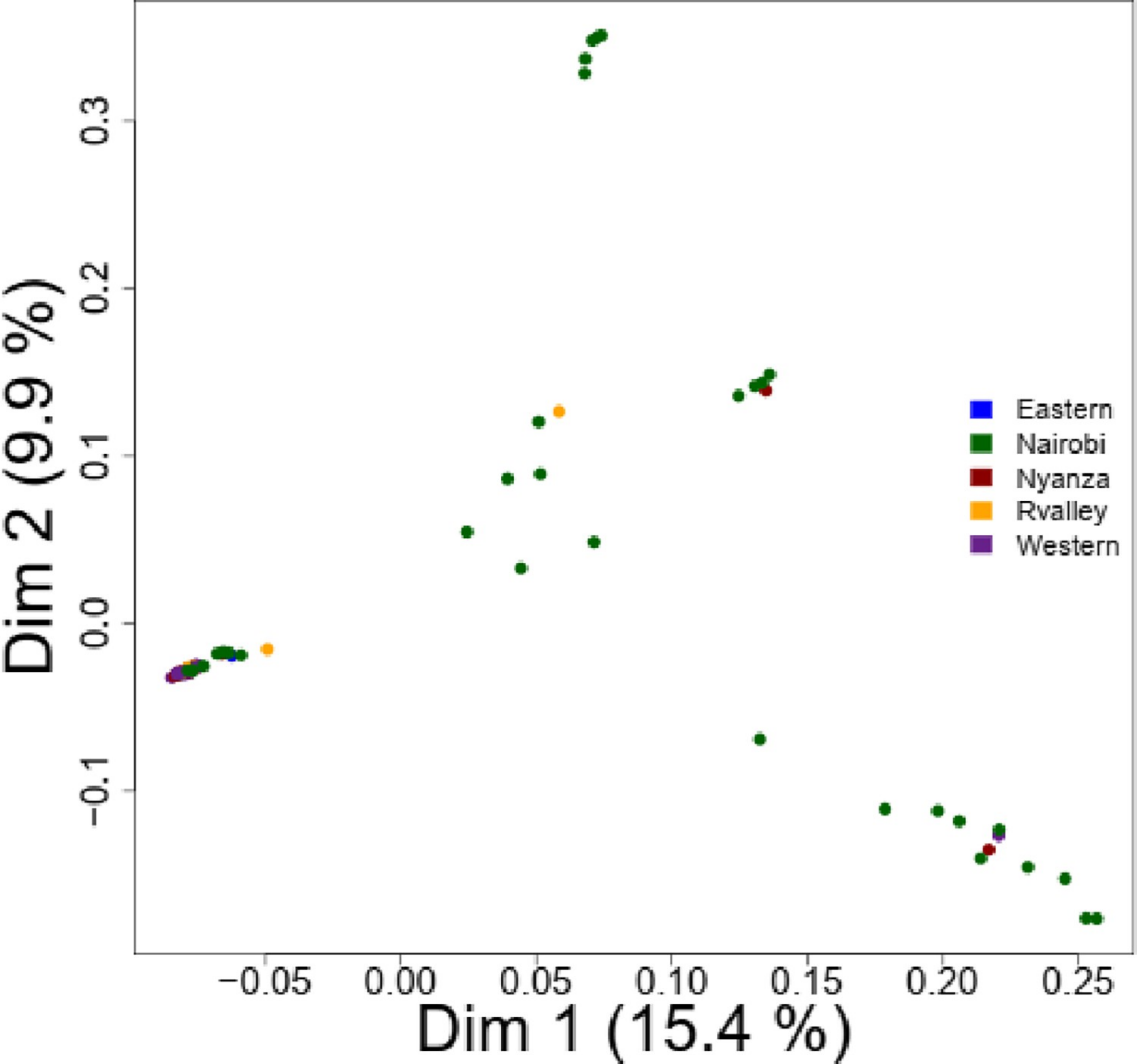
Principal component analysis (PCA) bi plot based on SNP data of 80 Kenyan *Crotalaria accessions*.

Genetic clustering between *Crotalaria* samples was inferred using DAPC. Thirty principal components were retained after the initial dimension reduction step which contained 95% of the total genetic variation. The four retained linear discriminant eigenvalues after cross-validation with the first three PCs accounted for 82.7% of the total variability (S5 Fig). Membership coefficients of the samples to each group were low, suggesting a high level of admixture and little population structure. A PCA plot however revealed a moderate level of population stratification, with the first two dimensions accounting for 27.3% of the observed variation (Fig 5). The principal coordinate analyses (PCoA) revealed a similar non distinct population stratification similar to that of DAPC. Based on the Hamming’s genetic distance, 73.2% of the variation was explained by the first two PCs.

The relationship between *Crotalaria* samples based on the geographical regions of origin, an aspect that cannot be inferred by DAPC was established using Correspondence Analysis (CA). Based on the CA plot for individual accessions, a wide distribution of the individual with tight overlaps was observed. Most of the accessions were located within the positive (upper) left quadrant, which comprised accessions from all sampled regions. However, there were single accessions among samples collected from Rift Valley, Nyanza and Eastern that did not fall in this quadrant. Nairobi accessions were distributed in all the four quadrants. The analysis revealed a level of population stratification (S6A Fig). The CA for all the *Crotalaria* populations in study revealed that the Western and Nyanza populations fall in the same (upper right) quadrant, while the Eastern and Rift Valley populations also fall in one (lower right) quadrant. Based on CA, the Western population was genetically closer to the Nyanza population, while the Rift Valley, Eastern and Nairobi *Crotalaria* populations were distantly related (S6B Fig).

**Fig 6:**
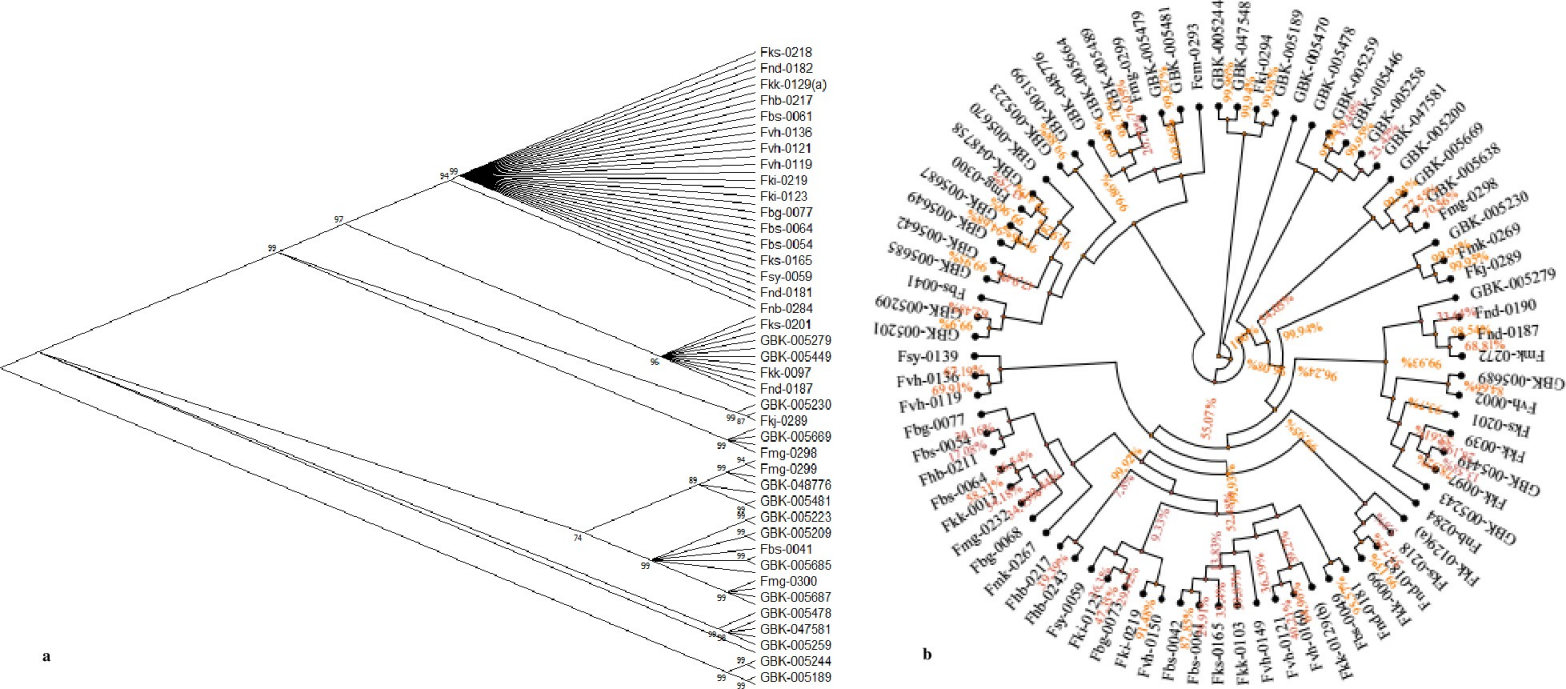
Phylogenetic analysis of 80 Kenyan *Crotalaria* accessions. (**a**) Phylogenetic tree generated using the Maximum Likelihood method and Tamura-Nei model using MEGA 11. (**b**) Consensus based phylogenetic tree generated using BEAST.

The maximum likelihood method of phylogenetic analysis clustered the 80 *Crotalaria* accessions into ten groups, depicting aspects of stratification. The largest clade consisted of the domesticated species *C*. *brevidens*, *C*. *ochroleuca* and *C*. *trichotoma*, while the second largest clade consisted of a mixture of both domesticated and wild accessions in the species *C*. *brevidens*, *C*. *trichotoma* and *C*. *intermedia* (Fig 6A). All the other clades consisted of wild accessions. The consensus BEAST based phylogenetic tree revealed three major clades, all consisting of a mixture of both wild and domesticated accessions (Fig 6B). This seemed to support the improper population stratification observed when using the distance based techniques especially DAPC.

## Discussion

Few studies have used genetic markers to study biodiversity in *Crotalaria spp*. These markers include expressed sequence tag-simple sequence repeat (EST-SSR), start codon targeted (SCoT) and internal transcribed spacer derived markers [2, 5, 6, 24]. Following advances in sequencing technology, next-generation sequencing and transcriptome analysis are the most preferred methods for studying plant diversity and availability of biological markers [49]. Despite the advances in technology, none of these techniques have been used in the study of genetic diversity in *Crotalaria*. The current study reports the first successful application of GBS for the study of *Crotalaria* species’ genetic diversity. The average number of reads per sample reported in this study is consistent with those reported by [50], who used a similar library preparation and sequencing technique to study diversity of cultivated Lentil (*Lens culinaris* Medik) species. The number of reads and the GC content of the obtained sequences in the current study, indicate that GBS is an appropriate molecular technique for germplasm characterization in *Crotalaria*. Based on the generated *de novo* assembly statistics, the NGSEP software was deemed adequate for the assembly, since it produced a moderate ratio of clustered to total reads in the assembled genome. This could be because it uses a hybrid approach of long and short reads, compared to tools which only rely on the long reads to generate an assembly [51].

Linkage disequilibrium in cross pollinated species such as *Crotalaria* is usually expected to decay at a short distance [52]. Although a fast decay was observed, LD decay in the present study depicted an extremely short decay. This could be due to high levels of recombination in a narrow gene pool in the domesticated species of *C*. *brevidens*, *C*. *ochroleuca* and *C*. *trichotoma*. The LD decay over a known genetic distance is important in determining the numbers and densities of markers necessary for breeding purposes [53]. Although there are no previous studies reported on the LD in *Crotalaria* species, the current study depicts a low level of LD. This could partly be due to the small population size involved in the study, or due to other aspects such as genetic drift which causes loss of rare allelic combinations [54]. The identification of outlier SNPs is important in genetic studies since these outliers correspond to regions of low recombination and high LD. Therefore, lack of many outlier SNPs in the current study could explain the observed LD patterns and the low population stratification. The genetic divergence aspects of the Kenyan *Crotalaria* species as observed in the study based on the Fst calculations reveal aspects of genome differentiation, which could be attributed to domestication. The observation of relatively high Fst values between domesticated and wild accessions and low values between purely domesticated or entirely wild accessions supports the aspect of genome differentiation, which has also been observed in cotton [55].

Single nucleotide polymorphism genotyping provides an appropriate and powerful phylogenetic analysis basis to study relatedness in plants and other organisms. To achieve accurate results for kinship estimation, it is necessary to have a reliable reference of allele frequencies in addition to having a large sample size [56]. A high proportion of loci with a MAF is always desired to achieve good pairwise relatedness estimates. Although in this study we reported 45.08% of SNPs with a MAF > 0.05, other factors such as the small sample size and the *de novo* assembly technique employed could have informed the low population stratification observed. Although GBS techniques are reliable and appropriate for genotyping individuals with a large numbers of SNPs, genotype call rates resulting from these techniques are low due to the low sequence depths involved [57]. Further, as an analysis strategy, SNPs are usually filtered to retain only those with sufficient depth which ends up reducing the number of SNPs. For example, in the current study, 434,274 SNPs were initially identified before filtering, which reduced the SNPs to 9820, potentially leading to the elimination of low-frequency SNP markers. The initially high number would be ideal to provide an accurate picture of the relatedness of the involved *Crotalaria* samples.

Differentiation in the loci with private alleles was seen in the five populations of Kenyan *Crotalaria* species involved in this study. The Nairobi sub-population had the highest number and range of loci with private alleles. This could be attributed to the fact that it had the highest number of accessions from different species considered in this study, hence making it the most genetically diverse sub-population compared to the other four sub-populations. Calculating private alleles has the advantage of providing information on alleles that exist in only that one sub-population. With 3,628 private alleles, the Nairobi sub-population had 3,446 more private alleles than the next most diverse sub-population (Western), portraying high genetic diversity among *Crotalaria* species from Nairobi. Similarly, based on the number of private alleles per population, the Nyanza (24) and Eastern (1) sub-populations had the least genetic diversity since they had the least private alleles after filtering. Private allele data is essential since it gives useful information on unique genetic variability in specific loci while identifying individuals/genotypes that might be used as parental lines in breeding programs to optimize allele richness in a population [58]. The genetic diversity analysis between the five subpopulations revealed there is a distinct genetic variation between Nairobi and western subpopulations. This implies that there are possible selection signatures that can be harnessed for breeding purposes towards improving *Crotalaria* species as alternative vegetables.

The effects of population structure on nucleotide polymorphism based on population genetic parameters especially the Tajima’s D revealed an overall low (negative) value. These low values suggest the presence of many rare alleles in the Kenyan *Crotalaria* population, which could have resulted from expansion of these populations after a bottleneck, positive selection or a selective sweep [59]. Similar negative Tajima’s D values have been observed in *Giardia duodenalis*, *Plasmodium falciparum* and *Brassica rapa* [60–62].

The generated CA bi-plots for individual samples revealed overlaps in samples, depicting a high level of relatedness in individual accessions, hence a high level of sharing genetic material. The primary axes of the high-dimensional space formed by the simultaneous inclusion of many conditions and their related samples are revealed via CA. The decrease in dimension allows data to be projected in two or three dimensions of maximum variance, indicating that two or more variables are related if they appear close to each other in the plot [63]. The low dimension percentages in the individual CA plots also supports this observation. Additionally, as revealed by the CA bi-plots for the entire population, the closeness in relatedness in Nyanza and Western accessions could be attributed to the geographical closeness of the two regions. Ordination techniques based on SNP markers have also been used to depict diversity in other crops such as blueberries, wheat, olive and other plants [12, 64, 65].

Dendrogram analysis based on Maximum Likelihood method and Bayesian clustering techniques revealed some level of population stratification, in Kenyan *Crotalaria* accessions. Although the number of clusters observed were few when using the Bayesian based clustering method compared to the maximum likelihood method, both techniques revealed some level of population admixture and stratification. The presence of structure in Kenyan *Crotalaria* germplasm is not surprising due to two main reasons. First, there has been continuous domestication of certain edible *Crotalaria* species in Kenya particularly in the Western region. With domestication, continuous selection for desirable traits in domesticated species narrows the gene pool, thereby introducing an aspect of population structure. Out of the 80 accessions used in this study, 40 belonged to three domesticated species *C*. *brevidens*, *C*. *ochroleuca* and *C*. *trichotoma* whose domestication could have introduced a high level of population structure. In China, domestication was found to impact the diversity and structure of *Saccharina japonica* populations [66]. Secondly, gene flow due to migration could have influenced population structure. In Kenya, communities living in the Western and Nyanza regions migrate with indigenous germplasm to urban areas, especially Nairobi [18]. This could be the reason why the Nyanza population was observed to be genetically closer to that of Nairobi and Western. Selection and genetic drift have been observed to cause population structure in other plant populations such as wheat [67, 68]. The clustering of wild accessions with domesticated accessions provides useful information for breeders and genetic conservation works of these species. It has been demonstrated that wild crop relatives ensure biological diversity in a domesticated crop’s gene pool, hence they are crucial in breeding and conservation works [69].

## Conclusion

The genetic diversity and population structure of wild and cultivated *Crotalaria* accessions from Kenya was investigated using GBS. Based on the SNP diversity in these accessions, three structured populations/clusters are postulated. These clusters did not associate with the regions of origin but contained individuals spanning different geographical locations. From the Fst statistics, there is moderate genetic drift among Kenyan *Crotalaria* accessions, suggesting a relatively moderate level of germplasm exchange between the different regions. Demographically, it could be concluded that Kenyan *Crotalaria* accessions are expanding after a bottleneck event, most likely, a diversity shrink due to continuous domestication or a selection sweep. Additionally, this study determined that the Nairobi *Crotalaria* population is the most diverse based on the richness of private alleles, due to minimal domestication and species diversity in this region. Although the concept of predictive breeding in plants such as *Crotalaria* is yet to be embraced, independent studies in these species are slowly contributing important information necessary for the actualization of this technique. The current study reports the first GBS-based SNP markers in *Crotalaria* species, an important step in predictive breeding and marker assisted selection. This coupled with other studies on phenotypic parameters in *Crotalaria* could provide the two most important forms of information necessary for creating models for genotypic effects and the development of breeding values for *Crotalaria* populations. Furthermore, results from this study could be a beginning point for the development of QTLs in *Crotalaria* species for different important traits in the species. This together with other techniques such as the advanced backcross QTL method can be used to introgress exotic genes form the wild accessions to the domesticated individuals.

## Supporting information

S1 Fig Heterozygosity levels in the Kenyan *Crotalaria* SNP data. a) Heterozygosity levels in all sites per population before data filtration, b) Heterozygosity levels in the segregating sites per population, c) Multi Locus Heterozygosity (MLH) per population and d) standardized Multi Locus Heterozygosity per population.
(TIF)

S2 Fig Transition vs transversion ratios of minor and major alleles before and after filtering in 80 Kenyan *Crotalaria* accessions. (TIF)

S3 Fig Hardy-Weinberg Equilibrium (HWE) test scores for the entire study of *Crotalaria* population and for the individual sub-populations. (TIF)

S4 Fig Spatial population clustering and Bayesian population assignment. (**a**) Population assignment probabilities bar plot based on STRUCTURE, (**b**) Population stratification and assignment base on LEA, (c) population membership based on TESS. Contour lines represent spatial position of genetic discontinuities.
(TIF)

S5 Fig Discriminant analysis of principal components (DAPC) plot. Low population stratification could be inferred from the plot.
(TIF)

S6 Fig Coordinate analysis (CA) plots based on Kenyan *Crotalaria* SNP data. (**a**) CA bi plot for individual *Crotalaria* accessions from the five sampled regions. (**b**) Population CA plot for *Crotalaria* accessions from the five sampled regions in Kenya.
(TIF)

S1 Table Information summary of the 80 *Crotalaria* samples from Kenya used in the study. (XLSX)

S2 Table Sequencing information summary for the 80 *Crotalaria* samples from Kenya used in the study. (XLSX)

S3 Table Clustering statistics of the *de novo* assembly of raw reads from 80 *Crotalaria* samples from Kenya based on the NGSEPcore software version 4.1.0. (XLSX)

S4 Table Population genetic test statistics for the 80 Kenyan *Crotalaria* samples used in the study. (XLSX)
