# Supplementary figures and images for: Genetic diversity and population structure of wild and cultivated Crotalaria species based on genotyping-by-sequencing
Source: PLoS One. 2022 Sep 1;17(9):e0272955. doi: 10.1371/journal.pone.0272955 (PMC9436042; doi:10.1371/journal.pone.0272955)

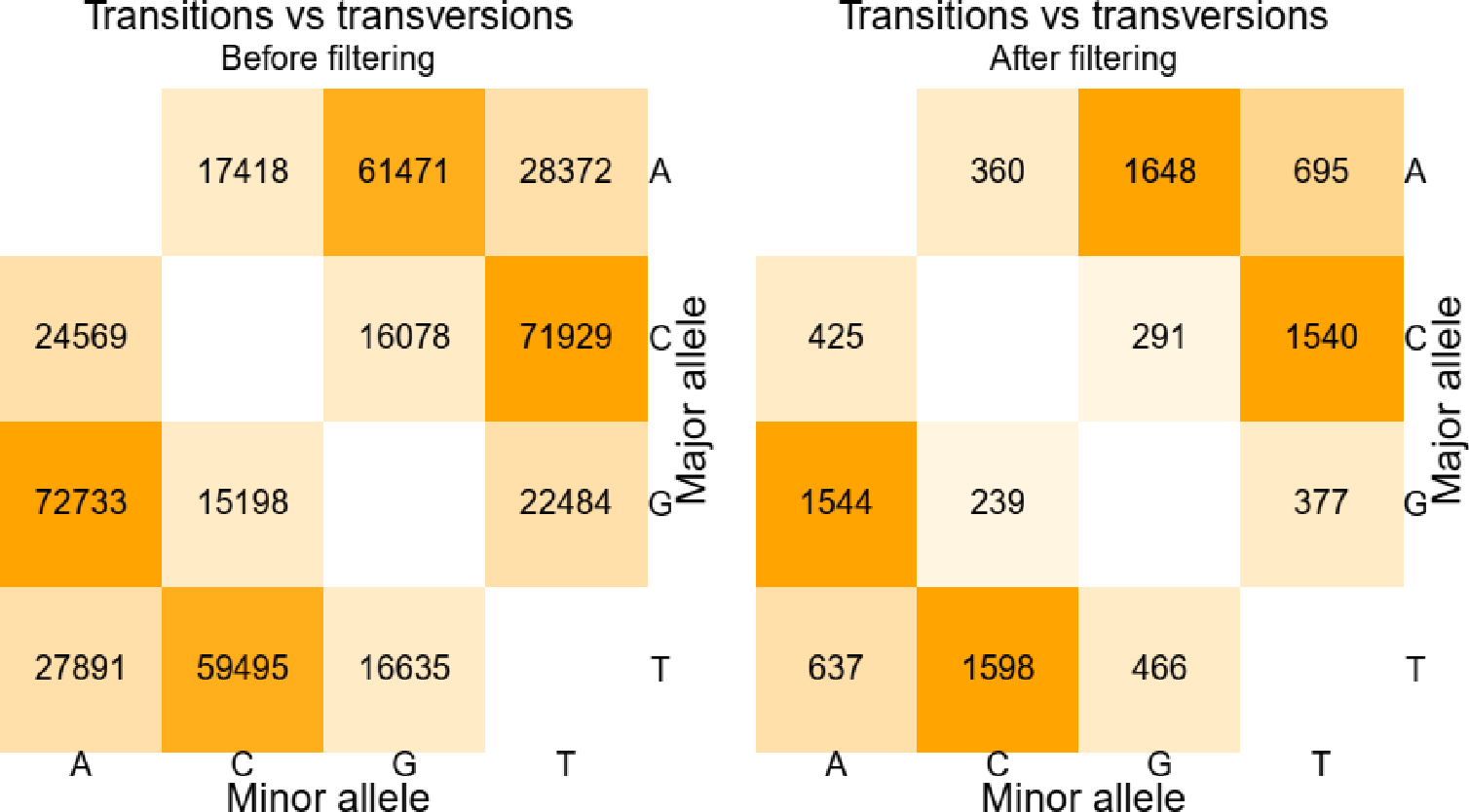

Supplement: S1 Fig — a) Heterozygosity levels in all sites per population before data filtration, b) Heterozygosity levels in the segregating sites per population, c) Multi Locus Heterozygosity (MLH) per population and d) standardized Multi Locus Heterozygosity per population. (TIF) [file pone.0272955.s001.tif]

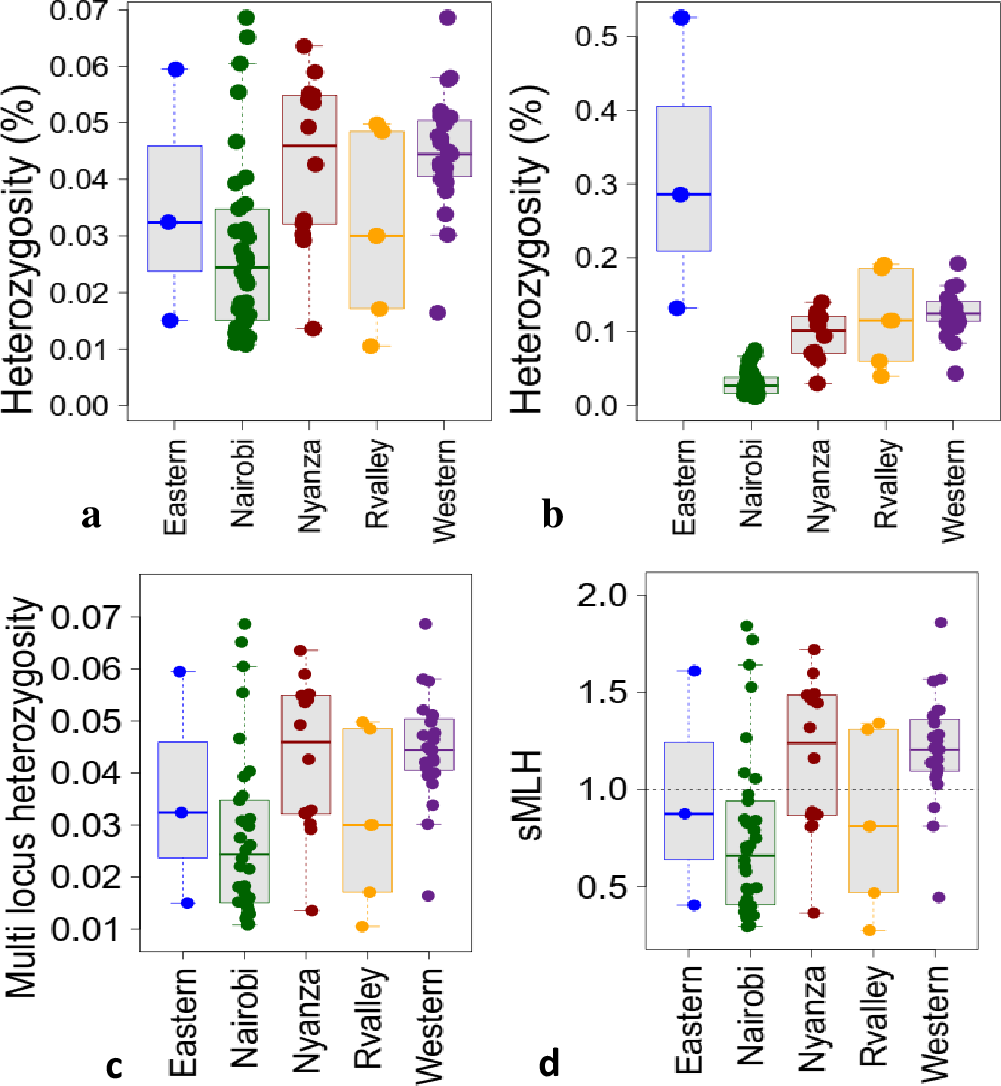

Supplement: S2 Fig — (TIF) [file pone.0272955.s002.tif]

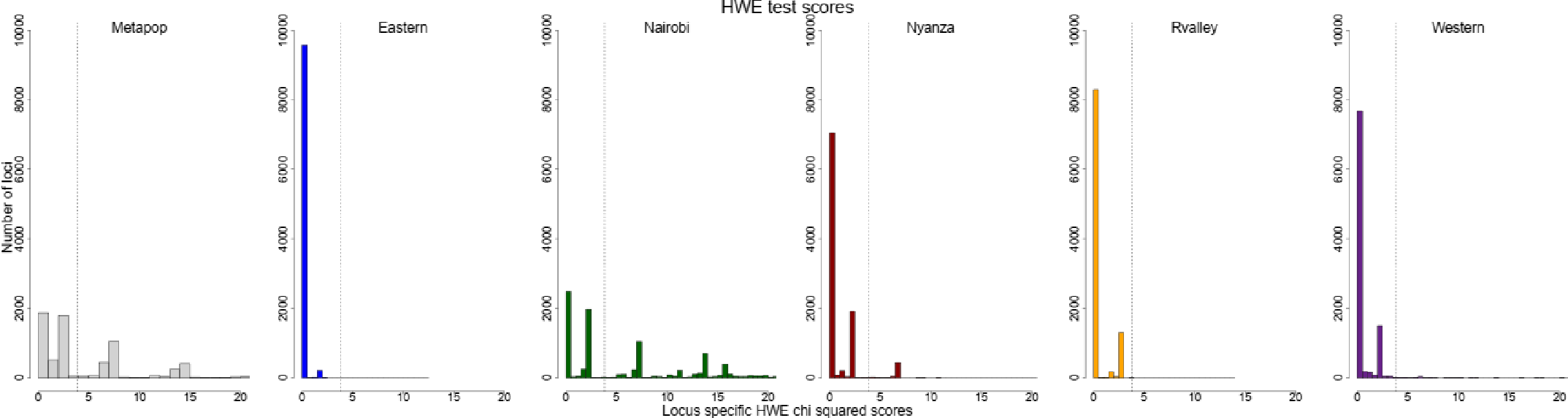

Supplement: S3 Fig — (TIF) [file pone.0272955.s003.tif]

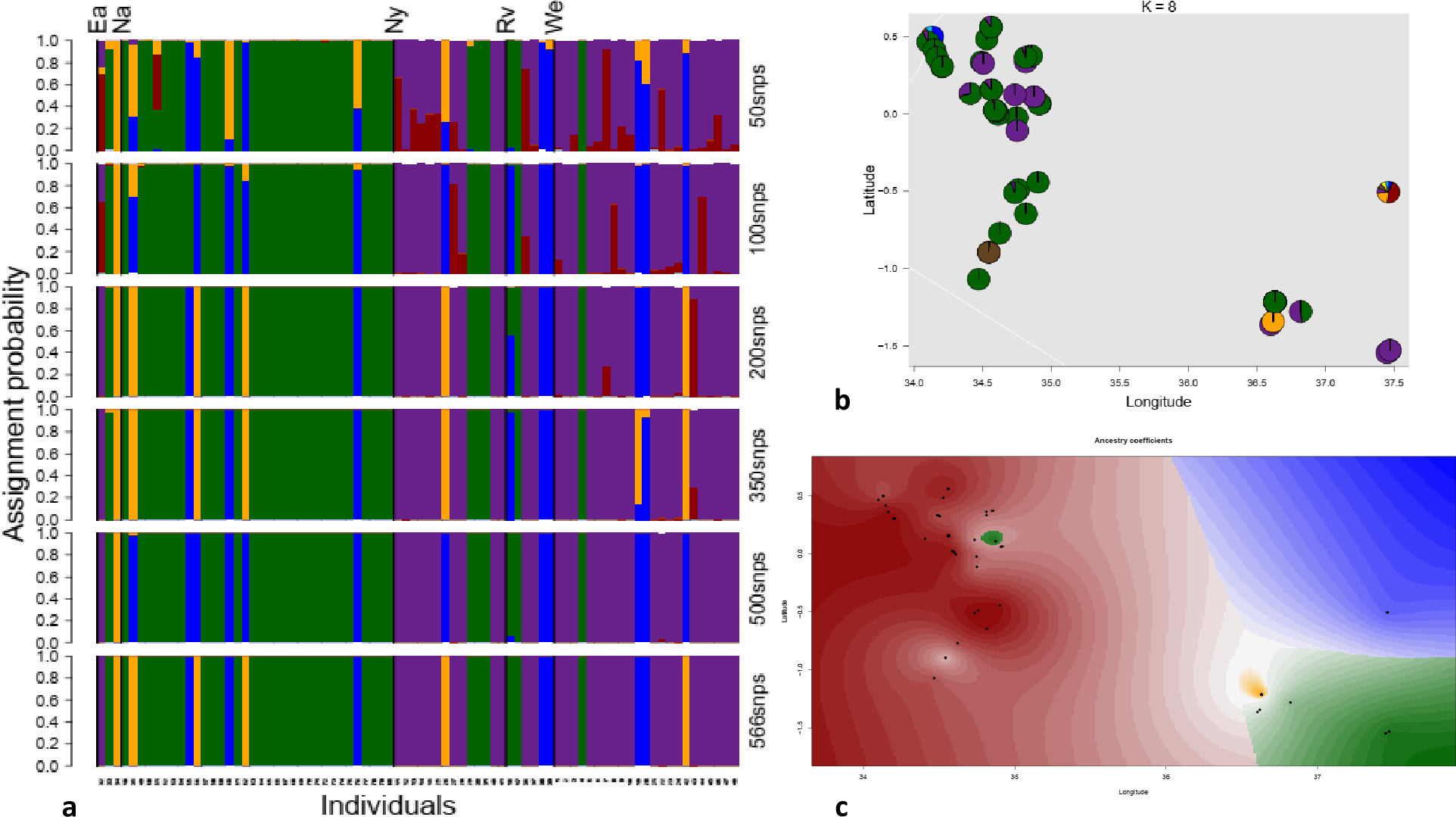

Supplement: S4 Fig — (a) Population assignment probabilities bar plot based on STRUCTURE, (b) Population stratification and assignment base on LEA, (c) population membership based on TESS. Contour lines represent spatial position of genetic discontinuities. (TIF) [file pone.0272955.s004.tif]

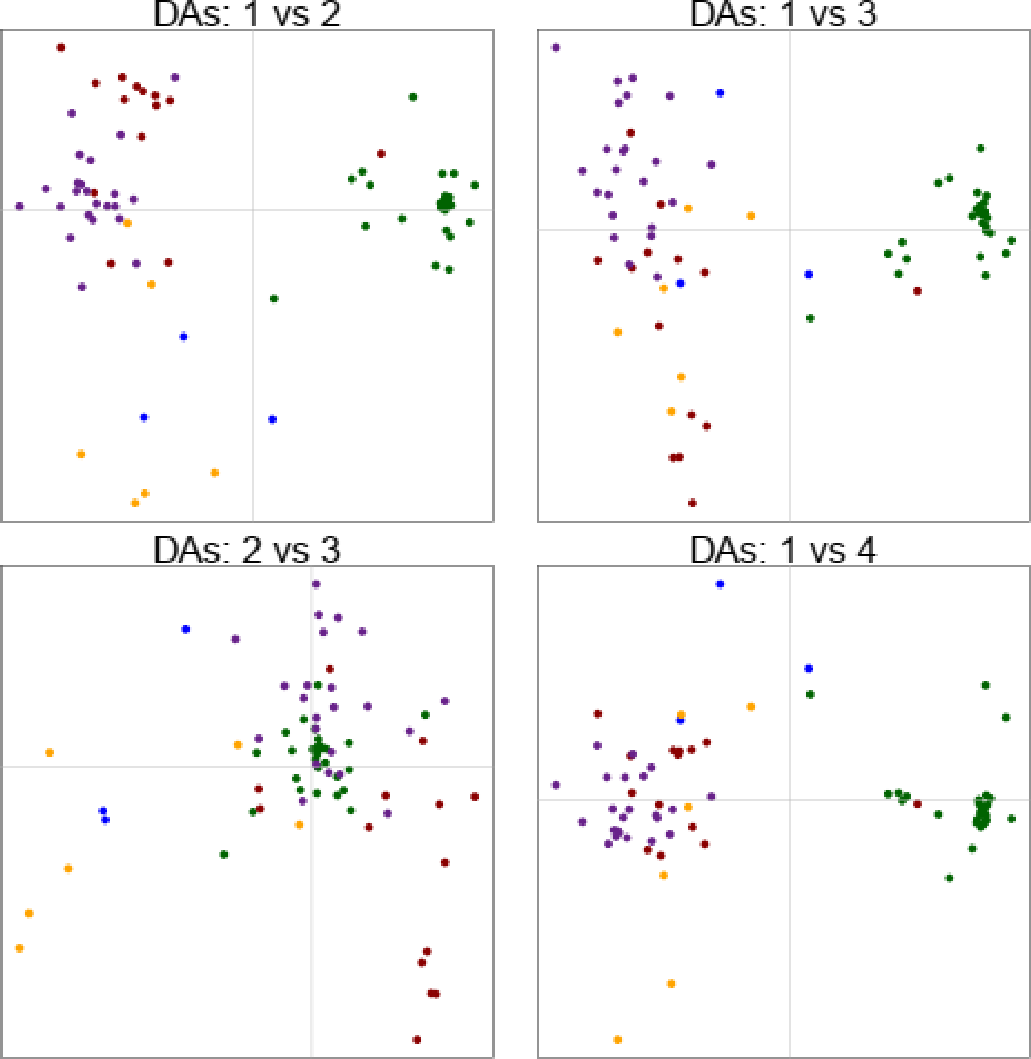

Supplement: S5 Fig — Low population stratification could be inferred from the plot. (TIF) [file pone.0272955.s005.tif]

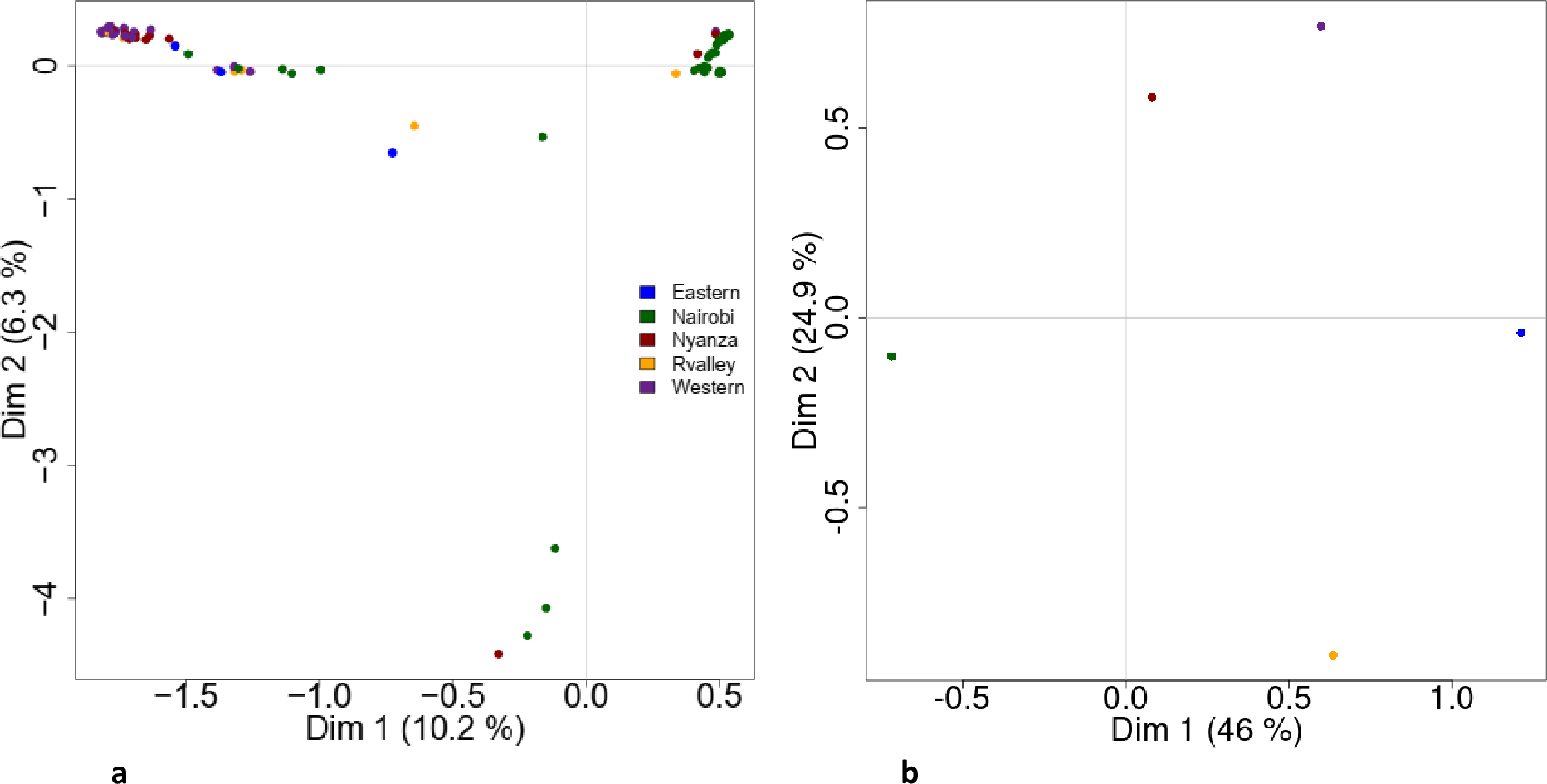

Supplement: S6 Fig — (a) CA bi plot for individual Crotalaria accessions from the five sampled regions. (b) Population CA plot for Crotalaria accessions from the five sampled regions in Kenya. (TIF) [file pone.0272955.s006.tif]
